# A Case of Post-operative Jaundice After Cardiac Surgery

**DOI:** 10.7759/cureus.35190

**Published:** 2023-02-19

**Authors:** Fortune O Alabi, Christopher O Alabi, Brent Waldon, Fred C Umeh, George Palmer

**Affiliations:** 1 Pulmonary Medicine, Critical Care and Sleep Medicine, Florida Lung, Asthma & Sleep Specialists, Orlando, USA; 2 Internal Medicine, HCA East Florida Westside Hospital/Northwest Hospital, Plantation, USA; 3 Internal Medicine, Florida Lung, Asthma & Sleep Specialists, Orlando, USA; 4 Cardiovascular Surgery, AdventHealth Orlando, Orlando, USA; 5 Pulmonary Medicine and Critical Care Medicine, Florida Lung, Asthma & Sleep Specialists, Orlando, USA

**Keywords:** hyperbilirubinemia, blood transfusion, tricuspid valve replacement, mitral valve replacement, valvular heart disease, cardio-pulmonary bypass surgery, post-cardiac surgery hyperbilirubinemia

## Abstract

Hyperbilirubinemia is a common gastrointestinal complication seen post-cardiac surgery. Here, we describe a case of a 72-year-old male with a past medical history of chronic obstructive pulmonary disease, chronic kidney disease (CKD), pulmonary hypertension, and valvular heart disease with severe aortic stenosis, severe mitral and tricuspid regurgitations who underwent elective aortic valve replacement (AVR), mitral valve replacement (MVR), and tricuspid valve (TV) repair; in addition, he required left thoracotomy for the repair of pulmonary artery perforation from a Swan-Ganz catheter that resulted in a large left pleural bleed. Post-operatively, he developed severe jaundice, which was predominantly conjugated that peaked at 24 mg/dL. He also required multiple blood products' transfusion in the perioperative period and was supported temporarily with hemodialysis for acute kidney injury superimposed on his CKD. He underwent extensive evaluation for jaundice, which included ultrasound of the liver, hepatobiliary iminodiacetic (HIDA) scan, and magnetic resonance cholangiopancreatography (MRCP), which were all normal. The patient eventually got better and was discharged from the hospital. The hyperbilirubinemia slowly got better without any specific therapy and on his follow-up visit to the office following discharge, his bilirubin level was found completely normalized.

Although most cases of post-cardiac surgery hyperbilirubinemia resolve without any specific therapy, the occurrence is not completely benign since it can increase morbidity and mortality. It is paramount that intensivists and cardiothoracic surgeons caring for these patients are aware of this occurrence to prevent unnecessary diagnostic evaluation. Most early cases of hyperbilirubinemia are transient and do not usually increase morbidity and mortality. In the late cases, infectious etiology resulting in sepsis needs to be entertained early and treated aggressively.

## Introduction

Hyperbilirubinemia (defined as having bilirubin concentration greater than 3 mg/dL) is a fairly common post-operative complication after cardiac surgery. It is also the most commonly reported gastrointestinal complication post-cardiac surgery. Post-cardiac surgery hyperbilirubinemia (PCSH) usually occurs early (mostly observed between the 2nd and 10th days), and peaks on average by the ninth day. In the vast majority of patients, post-cardiac surgery, hyperbilirubinemia is a self-remitting process, but it can occasionally lead to high mortality, especially when it occurs late or when the peak bilirubin is ≥25 mg/dL. The disorder is still largely unappreciated among cardiothoracic surgeons and intensivists taking caring of patients in post-operative units who have this condition.

In most cases, the management in hospitals is supportive once the diagnosis has been established and other causes of post-operative hyperbilirubinemia have been excluded. It is essential to avoid hepatotoxic medication in patients with PCSH as part of the conservative management.

## Case presentation

This is the case of a 72-year-old man with a relevant past medical history of severe aortic stenosis, severe mitral regurgitation, severe tricuspid regurgitation, and pulmonary artery hypertension. He was admitted to undergo aortic valve replacement (AVR) and mitral valve replacement (MVR) as well as a tricuspid valve (TV) repair. During admission, the patient reported that he was examined for abdominal pain by another emergency department four weeks earlier, where an ultrasound scan revealed multiple gallstones. No surgical intervention was required at that time. The patient also had a history of chronic obstructive pulmonary disease (COPD) on home oxygen, hyperlipidemia, tobacco use (smoked two packs per day for 40 years and stopped in year 2003), hypothyroidism, chronic kidney disease (CKD), and a history of metastatic bladder.

Intraoperatively, he underwent AVR, MVR, and TV repair. In addition, he needed a left thoracotomy for the repair of pulmonary artery perforation (caused by placing a Swan-Ganz catheter), which resulted in a large left pleural bleed. He had significant blood loss that necessitated transfusion of multiple blood products: he received six units of packed red blood cells (PRBCs), six units of fresh frozen plasma (FFP), four units of platelets, two units of cryoprecipitate, 2500 mL of cell saver intake, tranexamic acid, and protamine. The time taken for cardiopulmonary bypass (CPB) was 228 minutes and the time taken for the aortic cross-clamp was 189 minutes. Total time for surgery was 460 minutes, which included the time spent on performing a left thoracotomy.

Findings from the physical examination

Preoperatively, the patient was afebrile, and his blood pressure was 122/92 mmHg. His pulse was 82 beats per minute and oxygen saturation was 93% on 3 L/min oxygen. His chest examination revealed that breath sounds were diminished at bilateral lung bases. The patient had a 3/6 murmur and 1+ bilateral lower extremity edema. His chest x-ray indicated cardiomegaly without evidence of congestive heart failure (CHF) and chronic bilateral interstitial prominence. The patient's pertinent lab results before surgery are shown in Table 1.

**Table 1:** Summary of patient's pertinent pre-operative laboratory results

	Patient’s pre-operative findings	Normal range
White blood cell count	7.3 x 10^9^/L	4.0-10.5 x 10^9^/L
Hemoglobin	8.9 g/dL	11.2-15.7 g/dL
Hematocrit	32.6%	34.1%-44.9%
Platelets	152/μL	150-400/μL
Sodium	137 mmol/L	135-145 mmol/L
Potassium	4.9 mmol/L	3.5-5.2 mmol/L
Creatinine	2.57 mg/dL	0.43-1.13 mg/dL
Blood urea nitrogen	42 mg/dL	6-22 mg/dL
Total bilirubin	1.1 mg/dL	0.1-1.2 mg/dL
Alkaline phosphatase	69 u/L	30-120 u/L
Alanine transaminase	23 u/L	10-40 u/L
Aspartate aminotransferase	32 u/L	10-40 u/L

Hospital course

**Figure 1:**
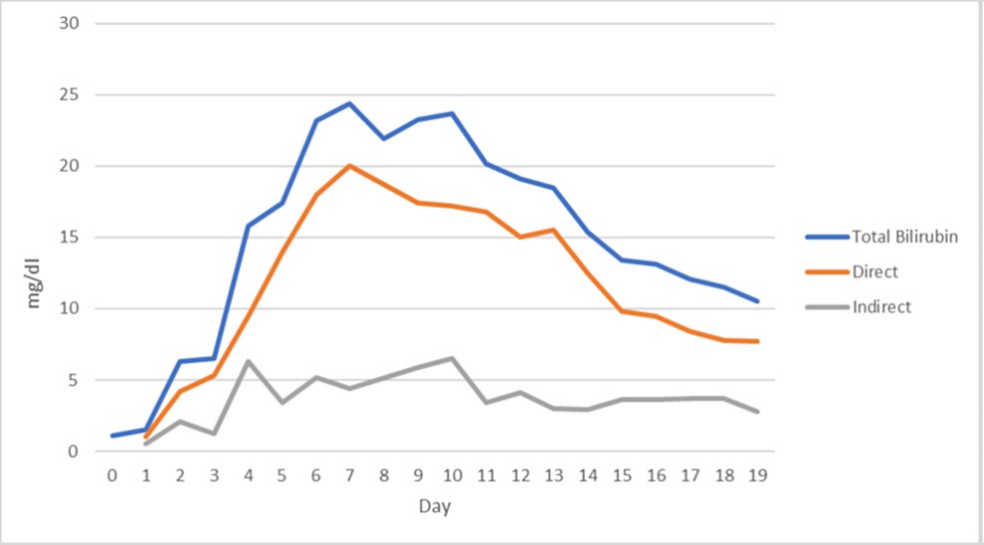
Chart of patient’s bilirubin (direct, indirect, and total) for the first 19 days after cardiac surgery

Postoperatively, the patient required vasopressor and inotropic support to maintain mean arterial pressure (MAP) greater than 60 mmHg. On the second post-operation day, he was observed to be clinically jaundiced. He also developed superimposed acute kidney injury (AKI) and required hemodialysis (HD) support during hospitalization. The post-operative results indicated renal failure and abnormal liver function tests (LFTs) (Figure 1).

The hepatitis profile, alpha-1 antitrypsin, and anti-smooth muscle antibody (ASMA) results were all normal. The results obtained from the ultrasound of the liver, hepatobiliary iminodiacetic acid (HIDA) scan, and magnetic resonance cholangiopancreatography (MRCP) were normal.

The bilirubin level started increasing on the second day post-operation and peaked at 23.7 mg/dL on the 10th day. The patient had predominantly conjugated hyperbilirubinemia, which accounted for 72% of the total bilirubin level. He underwent extensive diagnostic testing, the results of which came back normal. A diagnosis of hyperbilirubinemia after cardiac surgery was made after other causes were excluded. We also felt that the AKI could have contributed to the patient’s hyperbilirubinemia. The multiple blood transfusions he received in the intraoperative period could have also contributed to this diagnosis. All medications that could potentially be hepatotoxic were withheld. His kidney failure was supported with HD. He was discharged on day 18 and on his follow-up visit to the office (four weeks later), his bilirubin levels were found to be normal.

## Discussion

Post-cardiac surgery hyperbilirubinemia is defined as a bilirubin concentration greater than 3 mg/dL during the post-operative period, with an incidence of 3%-35% in most reported studies [1-4]. The significant variability in the reported incidences depends on many factors: the threshold of bilirubin increase that is used for the definition, the time frame post-surgery that is included for the surveillance, preexisting systolic heart failure and elevated right atrial pressure in the cohort, and percentage of patients in the cohort who underwent valve replacement or required blood transfusions. This entity was first published in 1967 by Lockey and colleagues. They reported an incidence of 13% amongst 233 patients who underwent open heart surgery [5].

Hyperbilirubinemia following cardiac surgery can be conjugated or unconjugated. Most cases are conjugated [1,4,6]. Few have mixed hyperbilirubinemia and some report predominant unconjugated hyperbilirubinemia [5]. The four categories of hyperbilirubinemia are (1) excessive production of bilirubin, (2) hepatocellular disorders, (3) cholestatic disorders, and (4) underlying liver disease. Category 1 usually results from hemolysis, resorption of hematoma, and excessive blood transfusion. Hemolysis from mechanical shearing forces secondary to CPB and synthetic valve also fall under this category. Category 2 includes ischemia, drugs, anesthetic agents, and viral hepatitis. Category 3 could be intrahepatic or extrahepatic cholestasis. The extrahepatic causes include stones, stricture, pancreatitis or cholecystitis, which can all easily be excluded with ultrasound or MRCP. The intrahepatic cholestasis includes post-cardiac surgery sepsis, or medications. The post-cardiac surgery hyperbilirubinemia also tends to occur early as against that occurring late, which is usually due to sepsis or medications. The post-cardiac surgery hyperbilirubinemia is usually accompanied by mild transaminases, and the alkaline phosphatase is only rarely significantly elevated. Ischemic hepatitis from low cardiac flow state, hypotension, or hypoxia is normally associated with very high levels of transaminases with elevated lactate dehydrogenase (LDH). The myriad causes of hyperbilirubinemia in the post-cardiac surgery state are the reason why it seems there is inconsistency in the predominant pattern of hyperbilirubinemia reported in the literature.

The type of cardiac surgery as well as the duration of aortic cross-clamp and the total time of the CPB are also important. Valve replacements have a higher incidence than cardiac bypass surgeries [7]. The mitral valve has a higher incidence of post-cardiac surgery bypasses than the aortic valve [5]. In a prospective observational study by Sharma et al., the incidence of post-operative jaundice in patients who underwent valve replacement was 36.2% compared to 12.7% with a coronary artery bypass graft (CABG) [8]. Although many studies have shown the correlation between cardiopulmonary bypass time (CPBT) and post-cardiac surgery, a causal relationship is yet to be established. In a study where thoracic surgery was compared to cardiac surgery (esophagectomy vs. open heart surgery), the incidence of post-operative hyperbilirubinemia was surprisingly the same [9].

The mechanism for PCSH is not well known but the cause is believed to be multifactorial. Biopsy reports have revealed the presence of centrilobular congestion, intrahepatic cholestasis, and mild necrosis. A diminished hepatic blood flow secondary to CPB has been implicated and this was also demonstrated in the study by Chetty et al. that included 10 volunteers with no history of underlying liver disease. In this study, the induction of anesthesia or initiation of mechanical ventilator did not decrease the hepatic blood flow, but the commencement of CPB reduced the hepatic blood flow significantly [10]. Pump-induced inflammation has been suggested to be a contributing factor [11]. Elevated right-sided pressure with increased right atrial pressure has been shown to correlate with the incidence of post-cardiac surgery jaundice [2,5,12]. This is probably due to hepatic congestion from the elevated right atrial pressure. A strong correlation has also been demonstrated with excessive blood transfusion during surgery as well as prolonged CPBT [1]. The presence of renal failure impairing the excretion of the conjugated bilirubin could also contribute to hyperbilirubinemia.

The evaluation of the post-operative patient with hyperbilirubinemia should begin with a detailed history of the patient, review of surgery notes, review of medications, laboratory results, imaging report, and time of the onset of jaundice, addressing the following questions: which type of bilirubin is elevated? What are the other liver chemistry levels? What were these values pre-operatively? Did the patient receive transfusions? Did the patient receive a cardiopulmonary bypass? What was the aortic cross-clamp time? What types of medication did the patient receive (including anesthesia)? Was there pre-existing liver disease? Was there pre-operative pulmonary hypertension or CHF? Was there pre-operative CKD?

## Conclusions

Hyperbilirubinemia is common following cardiac surgery and should be suspected in anyone who has had an early onset of post-operative jaundice after a cardiac surgery. Pre-operative risk factors include pulmonary hypertension, preexisting liver disease, CHF, and CKD. Intraoperative risk factors include multiple valve surgeries, prolonged cross-clamp time and prolonged CPBT, multiple blood transfusions, and hypotension. Prognosis is good in most patients, but it can result in prolonged hospitalization and can be occasionally associated with an increased risk of mortality.

## References

[1] Collins JD, Bassendine MF, Ferner R, Blesovsky A, Murray A, Pearson DT, James OF (1983). Incidence and prognostic importance of jaundice after cardiopulmonary bypass surgery. Lancet.

[2] Wang MJ, Chao A, Huang CH (1994). Hyperbilirubinemia after cardiac operation. Incidence, risk factors, and clinical significance. J Thorac Cardiovasc Surg.

[3] Farag M, Veres G, Szabó G, Ruhparwar A, Karck M, Arif R (2019). Hyperbilirubinaemia after cardiac surgery: the point of no return. ESC Heart Fail.

[4] Michalopoulos A, Alivizatos P, Geroulanos S (1997). Hepatic dysfunction following cardiac surgery: determinants and consequences. Hepatogastroenterology.

[5] Lockey E, McIntyre N, Ross DN, Brookes E, Sturridge MF (1967). Early jaundice after open-heart surgery. Thorax.

[6] Mastoraki A, Karatzis E, Mastoraki S, Kriaras I, Sfirakis P, Geroulanos S (2007). Postoperative jaundice after cardiac surgery. Hepatobiliary Pancreat Dis Int.

[7] Golitaleb M, Kargar F, Bakhshande Abkenar HB, Haghazali M, Gol Aghai F, Harorani M (2017). Hyperbilirubinemia after open cardiac surgery. Iranian Heart J.

[8] Sharma P, Ananthanarayanan C, Vaidhya N, Malhotra A, Shah K, Sharma R (2015). Hyperbilirubinemia after cardiac surgery: an observational study. Asian Cardiovasc Thorac Ann.

[9] Hosotsubo K, Nishimura M, Nishimura S (2000). Hyperbilirubinaemia after major thoracic surgery: comparison between open-heart surgery and oesophagectomy. Crit Care.

[10] Chetty G, Sharpe DA, Nandi J, Butler SJ, Mitchell IM (2004). Liver blood flow during cardiac surgery. Perfusion.

[11] Trauner M, Fickert P, Stauber RE (1999). Inflammation‐induced cholestasis. J Gastroenterol Hepatol.

[12] Bøhmer T, Elgjo K, Hovig T, Jacobsen CD, Bakken A, Skagseth E, Skrede S (1987). Postoperative jaundice in patients undergoing cardiac surgery. An incomplete cholestatic syndrome of multifactorial etiology. J Oslo City Hosp.

